# A systematic review of the responsiveness of lower limb physical performance measures in inpatient care after stroke

**DOI:** 10.1186/1471-2377-13-4

**Published:** 2013-01-10

**Authors:** Katharine Scrivener, Catherine Sherrington, Karl Schurr

**Affiliations:** 1 Bankstown-Lidcombe Hospital, 70 Eldridge Rd, Bankstown, NSW, 2200, Australia; 2 The George Institute for Global Health, The University of Sydney, PO Box M201, Missenden Road, Sydney, NSW, 2050, Australia

**Keywords:** Stroke, Responsiveness, Walking, Mobility, Measurement, Rehabilitation

## Abstract

**Background:**

Responsiveness refers to a measurement tool’s ability to detect change in performance over time. The aim of the review was to summarise studies of responsiveness of lower limb physical performance measures during inpatient care after stroke.

**Methods:**

A systematic literature review was conducted. Prospective studies that included participants with a diagnosis of stroke, were commenced in the acute or subacute phase of inpatient care and included a measure of a lower limb physical performance were included in this review.

**Results:**

Twenty-one studies met these inclusion criteria. A variety of measures were investigated including the Berg Balance Scale, various timed walking tests and the Rivermead Mobility Index. Ten of the included studies had small sample sizes (50 participants or less), 2 studies used a convenience sample rather than consecutive recruitment and 5 studies excluded potential participants with poor physical abilities at baseline. Responsiveness varied between and within studies but was generally large, Effect Size (ES) or Standardised Response Mean (SRM) > 0.8. Measures displaying large responsiveness included the twelve-minute walk test (SRM 1.90) and the Modified Rivermead Mobility Index (SRM 1.31) when re-measured at four weeks after stroke, and the Berg Balance Scale (ES 1.11) and Postural Assessment Scale for Stroke Patients (ES 1.12) when re-measured at approximately six months after stroke.

**Conclusion:**

Studies conducted to date have generally found physical performance measures after stroke to have large responsiveness i.e., to be able to detect changes. Further investigation of the responsiveness of measurement tools after stroke in larger prospective cohort studies is required.

## Background

Rehabilitation after stroke aims to optimise stroke survivors’ physical functioning including mobility. Physical performance measures involve measurement of mobility and other functional activities. These measures are often used to monitor progress in clinical settings and used in research studies to evaluate the effectiveness of rehabilitation programs. Many different measurement tools are available to evaluate physical performance in stroke survivors [1]. Clinicians and researchers need to decide on the best measurement tools to use after stroke. In order to make this decision each measurement tool’s reliability, validity and responsiveness must be considered [2,3].

Responsiveness is a measurement tool’s ability to detect change in performance over time [4]. If responsiveness of a measurement tool is low then even though a person’s physical performance may be improving the tool may not be sensitive enough to detect this change e.g. his/her score on a particular scale may not change. Different measurement tools may be more sensitive to change in different settings over different timeframes.

To date, responsiveness has not received the same attention in the literature as reliability and validity [5]. Despite this there has been a recent increase in research about the responsiveness of measures in stroke rehabilitation. This interest has lead to a number of papers being published summarising the responsiveness of one or more measurement tools. The aim of this systematic review was to summarise the current evidence about the responsiveness of lower limb physical performance measures during inpatient care after stroke. The purpose of this review is to provide clinicians and researchers access to a summary of the many measurement tools available and to assist their selection of the most relevant measurement tool.

The specific research question of this review was: how responsive are measurement tools that measure any aspect of lower limb physical performance in stroke survivors when the use of the measure commences in inpatient care, that is early after stroke?.

## Method

When designing and conducting this systematic review we followed the PRISMA guidelines for systematic reviews [6].

### Search strategy

A literature search was completed of Medline (via OvidSP, 1950 to April 2012), CINAHL (1981 to April 2012) and EMBASE (1980 to April 2012) databases for relevant articles. The search terms were stroke (or cerebrovascular accident or hemiplegia) and responsiveness and terms related to lower limb activities or physical therapy (including sitting or standing or standing up or sit to stand or balance or walking or gait or mobility or physiotherapy or physical therapy or rehabilitation). The first author reviewed titles and/or abstracts of displayed articles and determined relevance to the review. Full text copies of relevant articles were obtained. Reference lists were screened for identification of other relevant articles. Two known systematic reviews of measurement in neurological populations were screened for relevant articles [5,7]. Only articles written in English were included in this review. Any year of publication was included as restricted by each database’s availability.

### Inclusion criteria

Studies were included in the review if they: included only participants with a diagnosis of stroke; used a prospective design which involved initial measurement in inpatient care (acute hospital or subacute rehabilitation setting); involved a physical performance measure that related to a lower limb activity including seated reach, standing up, standing, balancing and walking.

A physical performance measure was defined as any measure that required the stroke survivor to actively move or participate. Each measure included use of the legs e.g. sitting, seated reach, moving from sitting to standing, balancing in standing and walking. If the first author was unfamiliar with a scale, a full copy of the scale was reviewed to check for inclusion of lower limb activities. Where upper limb activities were combined in the scales total score the scale was excluded from the review.

### Data extraction

Information about the study design, setting, participants and results were extracted by the first author and checked by the third author. Authors were contacted where there was information missing. When extracting data we looked for an Effect Size I (ESI) (also known as Cohen’s Effect Size, the difference between the mean baseline and follow-up scores divided by the standard deviation of baseline scores) or a Standardised Response Mean (SRM) (also known as Effect Size II, the ratio of observed change and its standard deviation, which therefore reflects the variability of change). However if an ESI or SRM was not calculated then, where possible, we calculated one from available data. We considered Effect Size/SRMs of 0.20 to 0.50 be small, 0.50 to 0.80 to be moderate and > 0.8 to indicate be large [4]. A recent consensus statement indicated that any statistical measure of change, including looking at the difference between before and after measures could be used to measure responsiveness [8]. Consequently if any statistical measure of change was used the article was included in this review.

### Assessing risk of bias

The risk of bias was evaluated for each study using the recent consensus statement regarding design of responsiveness studies as a guideline [8]. In particular we examined each studies sample size and the method used to select participants.

## Results

### Flow of studies through the review

The electronic search identified 189 articles. After screening all titles and abstracts, 26 articles were identified but after reviewing the full text, five of these were excluded from the review. The main reasons for exclusion was that the measurement tool involved an upper limb component or the population sample included people with a diagnosis other than stroke. A systematic review of the Berg Balance Scale was identified in the search and its reference list was also screened, identifying one extra article that had not been found in the electronic search [9,10]. When the reference lists of the two previously identified systematic reviews were screened, one additional article was identified and included in the review [11].

Upon reviewing the reference lists and abstracts of potentially relevant papers, from the 21 articles identified by this search, no extra articles were found to be relevant.

### Characteristics of included studies

The 21 included studies involved 1,101 participants (some of the included studies reported data for the same participants, where this was the case the participant was counted only once). A variety of measurement tools captured a number of lower limb skills ranging from seated reach to standing balance and walking. A summary of the studies is presented in Table 1. Additional information was obtained from the author of one study [12]. An Effect Size was calculated in one study where data was available but an Effect Size was not included in the original article [13].

**Table 1:** Summary of studies investigating the responsiveness of physical performance measures during inpatient care after stroke, n = 21

**Activity**	**Measure**	**Study**	**Setting**	**Inclusion timeframe post-stroke**	**n**	**Timeframe of reassessment, Result**
Seated Reach	Modified Functional Reach Test	Katz-Leurer 2009 [14]	Inpatient rehabilitation	14-21 days post stroke	n = 35	6 wks, Reach direction:
						Paretic side, ES = 0.80
						Forward, ES = 0.60
						Non-paretic side, ES = 0.57
Balance	PCBS	Pyoria 2005 [15]	Acute neurological ward	7 days post stroke	n = 50	7-120 days, p <0.001
					n = 42	120–360 days, p >0.05
					n = 42	7–360, p <0.001
	BBS	Salbach 2001 [16]	Acute care hospital	<8 days (as soon as ambulatory)	n = 50	4 wks, SRM=1.04
		Stevenson 2001 [10]	Stroke Unit (rehabilitation)	Admission, mean 30.3 days (SD 23.3)	n = 45	1-2 wks, p < 0.001
		Mao 2002 [17]	Hospital inpatients	14 days post stroke	n = 110	14 to 30 days, ES = 0.80
					n = 93	30 to 90 days, ES = 0.69
					n = 80	90 to 180 days, ES = 0.40
					n = 93	14 to 90 days, ES = 1.07
					n = 80	14 to 180 days, ES = 1.11
		English 2006 [11]	Inpatient rehabilitation	Within 1 wk of admission	n = 61	Within 1 wk of discharge (mean time admission to discharge 56.4+/− 38.1 days), ES = 1.01
		Wood-Dauphinee1996 [18]	Acute hospital inpatients	<2 wks post stroke	n = 60	2wks to 6wks, ES = 0.66, SRM = 0.81
						6wks to 12wks, ES = 0.25, SRM = 0.69
						2wks to 12wks, ES = 0.97, SRM = 1.08
	BBS-3P	Wang 2004 [19]	Hospital inpatients	14 days post stroke	n = 202	14 to 30 days, SRM = 0.82
					n = 167	30 to 90 days, SRM = 0.70
					n = 167	90 to 180 days, SRM = 1.11
	SFBBS	Chou 2006 [20]	Hospital inpatients	14 days post stroke	n = 81	14 to 90 days.
						14-item BBS, ES = 0.85
						7-item BBS, ES = 0.78
						6-item BBS, ES = 0.78
						5-item BBS, ES = 0.70
						4-item BBS, ES = 0.69
	FM-B	Mao 2002 [17]	Hospital inpatients	14 days post stroke	n = 110	14 to 30 days, ES = 0.82
					n = 93	30 to 90 days, ES = 0.63
					n = 80	90 to 180 days, ES = 0.33
					n = 93	14 to 90 days, ES = 1.06
					n = 80	14 to 180 days, ES = 1.14
	SBM	Chien 2007 [21]	Inpatient rehabilitation	< 3 months (mean 41.1 +/− 17.6)	n = 40	2wks,
						Equilibrium score, ES = 0.63
						Limits of stability time, ES = 0.27
						Limits of stability path, ES = 0.33
						Weight shifting, ES = 0.04-0.29
Walking	10mWT	Goldie 1996 [22]	Inpatient rehabilitation	Start of rehabilitation, median 16.5 days of hospitalization	n = 42	8 wks, ES=1.17
		Salbach 2001 [16]	Acute care hospital	<8 days (as soon as ambulatory)	n = 50	4wks, Comfortable pace, ES = 0.74, SRM = 0.92
						4wks, Maximum pace, ES = 0.55, SRM = 0.83
	5mWT	Salbach 2001 [16]	Acute care hospital	<8 days (as soon as ambulatory)	n = 50	4wks, Comfortable pace, ES = 0.83, SRM = 1.22
						4wks, Maximum pace, ES = 0.66, SRM = 1.00
		English 2006 [11]	Inpatient rehabilitation	Within 1 wk of admission	n = 61	Within 1 wk of discharge (mean time admission to discharge 56.4+/− 38.1 days), ES = 0.81
	2MWT	Kosak 2005 [23]	Inpatient rehabilitation	28 days (+/− 34 days)	n = 18	4 wks, SRM=1.34
	6MWT	Kosak 2005 [23]	Inpatient rehabilitation	28 days (+/− 34 days)	n = 18	4 wks, SRM=1.52
	12MWT	Kosak 2005 [23]	Inpatient rehabilitation	28 days (+/− 34 days)	n = 18	4wks, SRM = 1.90
	FAC	Kollen 2006 [24]	Hospital inpatients	<14 days (mean days post stroke = 8.2 (SD 2.8)	n = 101	4-26wks (weekly in weeks 1–10, fortnightly in weeks 10–20, once at week 26).
						Responsiveness ratios based on a 10% MCID exceeded the smallest detectable difference and ranged from 4.36 to 17.70.
		Mehrholz 2007 [25]	Early rehabilitation centre	30-60 days	n = 55	2wks SRM=1.016
						2wks to 4wks SRM = 0.842
						4wks to 6mths SRM = 0.699
Multiple	Modified EFAM	Liaw 2005 [26]	Subacute stroke inpatients	Admission	n = 40	Discharge, SRM = 1.1
	MAS	English 2006 [11]	Inpatient rehabilitation	Within 1 wk of admission	n = 61	Within 1 wk of discharge (mean time admission to discharge 56.4+/− 38.1 days).
						Item 1, ES = 1.03
						Item 2, ES = 0.74
						Item 3, ES = 0.61
						Item 4, ES = 0.85
						Item 5, ES = 1.02
	RMA	Kurtais 2009 [12]	Inpatient rehabilitation	Median 2 months (mean 5.6, SD 11.2, range 0.5 to 78 months)	n = 107	Discharge.
						RMA – gross function, ES = 0.51, SRM = 0.83
						RMA – leg and trunk, ES = 0.45, SRM = 0.86
	RMI	Hsueh 2003 [27]	Hospital inpatients	14 days	n = 43-51	30 days, SRM=1.14
						90 days, SRM=0.86
						180 days, SRM=0.24
		Hsieh 2000 [13]	Inpatient rehabilitation	Admission to rehabilitation, median 24 days post stroke (range 7 – 53 days)	n = 38	Discharge, ES = 1.28
Multiple		Franchignoli 2003 [28]	Inpatient rehabilitation	Admission	n = 73	5 wks, ES = 0.89
	MRMI	Hsueh 2003 [27]	Hospital inpatients	14 days	n = 43-51	30 days, SRM=1.31
						90 days, SRM=0.83
						180 days, SRM=0.20
		Lennon 2000 [29]	Inpatient rehabilitation	Admission	n = 16	Discharge, ES=1.15
	PASS	Wang 2004 [19]	Hospital inpatients	14 days post stroke	n = 202	14 to 30 days, SRM = 0.84
					n = 167	30 to 90 days, SRM = 0.65
					n = 167	90 to 180 days, SRM = 1.02
		Mao 2002 [17]	Hospital inpatients	14 days post stroke	n = 110	14 to 30 days, ES = 0.89
					n = 93	30 to 90 days, ES = 0.64
					n = 80	90 to 180 days, ES = 0.31
					n = 93	14 to 90 days, ES = 1.07
					n = 80	14 to 180 days, ES = 1.12
		Chien 2007 [21]	Inpatient rehabilitation	< 3 months (mean 41.1 days +/− 17.6)	n = 40	2wks, ES = 0.41
	PASS–3P	Wang 2004 [19]	Hospital inpatients	14 days post stroke	n = 202	14 to 30 days, SRM = 0.86
					n = 167	30 to 90 days, SRM = 0.67
					n = 167	90 to 180 days, SRM = 1.04
	6 SFPASS	Chien 2007 [30]	Hospital inpatients	14 days post stroke	n = 262	30 days, ES = 0.43-0.44
	PASS-TC	Wang 2005 [31]	Hospital inpatients	14 days post stroke	n = 246	14 to 30 days, SRM = 0.65
					n = 203	30 to 90 days, SRM = 0.42
					n = 189	90 to 180 days, SRM = 0.02

Abbreviations: *ES* = Effect Size, *SRM* = Standardized response mean, wks = weeks, *BBS-3P* = Berg Balance Scale three point, *BBS* = Berg Balance Scale, *SFBBS* = Short Form Berg Balance Scale, *FM-B* = balance subscale of the Fugl-Meyer test, *SBM* = Smart Balance Master, *10mWT* = ten metre walk test, 5*mWT* = five metre walk test, *2MWT* = two minute walk test, *6MWT* = six minute walk test, *12MWT* = twelve minute walk test, *FAC* = Functional Ambulation Category, *EFAM* = Emory Functional Ambulation Profile, *MAS* = Motor Assessment Scale, *RMA* = Rivermead Motor Assessment, *RMI* = Rivermead Mobility Index, *MRMI* = Modified Rivermead Mobility Index, *Motor-FIM* = Motor component of the Functional Independence Measure, *PASS* = Postural Assessment Scale for Stroke Patients, *6 SFPASS* = 6-item Short Form Postural Assessment Scale for Stroke Patients, *PASS-3P* = Three Point Postural Assessment Scale for Stroke Patients, *PASS-TC* = Postural Assessment Scale for Stroke Patients Trunk Control, *PCBS*= Postural Control and Balance for Stroke.

Most of the included studies were prospective cohort studies. Limitations of studies include ten out of the 21 studies having small sample sizes, 16 to 50 participants [10,13,14,16,21-23,26,27,29], use of a sample of convenience not consecutive stroke survivors [15,23] and having significant exclusion criteria e.g. excluding stroke survivors based on their physical abilities on admission [11,14,16,22,30]. Six studies in the review investigated the responsiveness of different measures in the same cohort of stroke survivors; in this case data were collected as part of a larger prospective study [17,19,20,27,30,31].

### Time after stroke

The time post stroke varied across the studies from 7 days to 2 months. Some studies included participants on admission to inpatient rehabilitation. In many of the studies occurring in subacute rehabilitation, the exact time post stroke onset was not indicated [11,16,22,26,28].

### Timeframe of follow up

The time between initial measurement and follow up varied greatly, from one to two weeks to 360 days after the initial measurement. A number of studies followed participants from admission until discharge from hospital [11-13,26,29]; consequently the exact timing between measures was unknown.

### Setting

All participants’ measurements commenced early in an inpatient hospital admission. Eleven of the studies specified they were conducted in inpatient rehabilitation. Three were conducted on acute hospital wards. The remaining seven studies indicated that they were conducted in an inpatient hospital setting but further details were not provided.

### Outcome measures

The measurement tools investigated varied. The Berg Balance Scale (BBS) was most investigated, with five studies included in this review investigating the full scale and two investigating a shortened version. For further details of measures investigated refer to Table 1.

### Responsiveness of measures of lower limb physical performance

#### Seated reach

The Modified Functional Reach Test towards the paretic side showed good responsiveness (ES 0.80) [14]. This reach direction was more responsive than the reach forward or towards the non-paretic side (ES 0.60 and 0.57 respectively).

#### Balance

The Berg Balance Scale demonstrated varied responsiveness depending on the study and time of measure post stroke (ES 0.40 – 1.11, ES > 1 in 3/9 measures and SRM 0.67 – 1.29, SRM > 1 in 2/4 measures) [11,16-19]. A three item short form of the BBS was shown to have similar results (SRM 0.70 and 1.11) [19].

#### Walking

The two-minute walk test, 2MWT (SRM 1.34) [23], six-minute walk test, 6MWT (SRM 1.52) [23] and twelve-minute walk test, 12MWT (SRM 1.90) [23] were highly responsive to change. However, they were investigated in a small study of only 18 participants. The 5 m (ES 0.66-0.81 and SRM 1.00-1.22 > 1 in 2/2 measures) [11,16] and 10 m (ES 0.55-1.17, ES > 1 in 1/3 measures and SRM 0.83-0.92) [16,22] walk tests demonstrated variable responsiveness.

#### Multiple

A number of studies investigated tools that rated subjects based on their ability to complete a number of activities from sitting to walking. Highly responsive measures included the Rivermead Mobility Index (ES 0.89-1.28, ES > 1 in 1/2 measures and SRM 0.24-1.14, SRM > 1 in 1/3 measures) [13,27,28], the Modified Rivermead Mobility Index (ES 1.15 and SRM 0.20-1.31, SRM > 1 in 1/3 measures) [27,29,32] and the Modified Emory Functional Ambulation Profile (SRM 1.1) [26]. The lower limb items of the Motor Assessment Scale (MAS) varied in their responsiveness (ES 0.61-1.03, ES > 1 in 2/5 items) [11]. The most responsive items were item 1; supine to side lying (ES 1.03) and item 5; walking (ES 1.02) [11]. The Postural Assessment Scale for Stroke patients (PASS) demonstrated variable responsiveness (ES 0.31-1.12, ES > 1 in 2/6 measures and SRM 0.84-1.02, SRM > 1 in 1/3 measures) [17,19,21,33]. It was most responsive when measured at later time periods after stroke e.g. comparing measures taken at 90 and 180 days post stroke (SRM 1.02) [19] and comparing measures taken at 14 and 180 days post stroke (ES 1.12) [17]. Full details of the responsiveness of each measurement tool, can be seen in Table 1.

## Discussion and conclusion

This systematic review summarised current studies relating to responsiveness of lower limb physical performance measures after stroke. This review demonstrated the variability in the responsiveness of these measures. Within the first four weeks after stroke the measures achieving an ES or SRM of greater than one were the Berg Balance Scale [16], 5-metre walk test [16], 2, 6 and 12 min walking tests [23], the Functional Ambulation Category [25] and the modified Rivermead Mobility Index [27]. When the follow up period was longer, for example more than three months after stroke, the measures achieving an ES or SRM of greater than one were the Berg Balance Scale [17,18] (including modified versions [17,19]) and the Postural Assessment Scale for Stroke [17,19] (including a modified version [19]).

This review confirms that responsiveness is specific to the population being investigated and the timeframe of measurement [4]. For example, the Effect Size for the ten-metre walk test (10mWT) varied from moderate (ES = 0.55 to 0.74) in one study conducted in an acute hospital with a measurement period of four weeks, to large (ES = 1.17) in another study conducted in rehabilitation with a measurement period of eight weeks. This makes identification of responsive measurement tools more challenging, as it is often not appropriate to compare results across studies.

The review was designed to assist clinicians and researchers to select the most appropriate measurement tool for use in their setting or trial. When making this decision a number of factors need to be considered. Firstly, they need to identify studies with a similar setting to their own in Table 1 of the review. For example will the measurement tool be used in an acute ward or rehabilitation unit? Secondly, when after stroke will they first measure the stroke survivors’ performance? Table 1 includes the timeframe when studies reported the initial measurement. Thirdly, they need to consider when they plan to re-measure the stroke survivors’ performance and find studies in Table 1 of the review that have assessed responsiveness with similar re-measurement periods. It is important to recognise that the responsiveness of the same measure can vary greatly if measured two weeks or six months later.

To our knowledge this is the first systematic review to focus on responsiveness of measurement tools in the stroke population. There have been several other reviews of mobility measurement tools in general neurological populations. These reviews found responsiveness to be rarely investigated [5,7]. The mobility measures found to be responsive for use in general neurological populations were the 5 m and 10 m walking tests, the 6MWT, the BBS and the Rivermead Mobility Index [5,7]. These measures were also included in our review and shown to be responsive in a stroke specific population.

The results of our systematic review need to be interpreted with caution due to the limitations of the included studies. For example, the two, six and 12-min walk tests demonstrate large Effect Sizes, however were investigated in a small study of just 18 participants [23]. Perhaps if they were investigated in a larger cohort the results would be different. In the review there were nine other studies with sample sizes containing less than 50 participants [10,13,14,16,21-23,26,27,29].

Moreover, a number of studies included in the review included data from the same cohort of stroke survivors [17,19,20,27,30,31]. As described above, responsiveness is specific to the sample being investigated. Consequently, it may appear in this review that more samples of stroke survivors have been assessed than is actually the case. Two studies in the review used a sample of convenience and not consecutive stroke survivors [15,23]. This study design may contain significant bias as subjects may be chosen as they are determined to be likely to respond to treatment.

In conclusion, this review has demonstrated the responsiveness of lower limb physical performance measures. The responsiveness of these measures was generally large. However, further systematic investigation of the responsiveness of measures in larger prospective cohort studies is required.

## Competing interests

The authors declare no competing interests.

## Authors’ contributions

KScr participated in the study design, carried out the electronic search, extracted data from trials for the review and drafted the manuscript. CS initiated the study design and participated in manuscript editing and writing. KSch participated in the study design, extracted data from trials for the review and participated in manuscript editing and writing. All authors read and approved the final manuscript.

## Pre-publication history

The pre-publication history for this paper can be accessed here:

http://www.biomedcentral.com/1471-2377/13/4/prepub
